# Ringworm: Its Nature, Diagnosis, and Treatment

**Published:** 1897-01-16

**Authors:** David Walsh

**Affiliations:** Physician Western Skin Hospital, London; Clinical Assistant Blackfriars Skin Hospital.


### RINGWORM: ITS NATURE, DIAGNOSIS, AND TREATMENT.

By DAVID WALSH, M.D.Edin., Physician Western Skin Hospital, London; Clinical Assistant Blackfriars Skin Hospital.

(*Continued from page* 248.)

**Forms of Ringworm.**—Ringworm may attack any part of the surface of the body. When it gives rise to spreading rings or ovals of various sizes on smooth skin it is known as (*a*) tinea circinata. Or it may attack the hairy parts; on the beard it is known as (*b*) tinea barbæ or tinea sycosis, and on the scalp as (*c*) tinea tonsurans. The meaning of tinea is a moth-worm, applied to this disease from the supposed analogy of a gnawing worm.

**Diagnosis.**—Tinea of the body, or tinea circinata, may be recognised as a red circular or oval desquamating patch, which has developed steadily from a small red point of origin. The edge of the patch is red, raised, and sharply marked, while its centre is discoloured and dotted with fine scales, or the whole surface may become inflamed. There may be one or more such patches, often on uncovered parts, as the face, neck, or hands, or several of the rings may run together and form a festoon or figure-of-eight pattern. This circinate form is often caught from dogs or horses, and may attack folk of all ages. This latter fact separates it sharply from the small-spored ringworm of the scalp, which is essentially a disease of children, and is of doubtful occurrence after puberty.

1. **Tinea circinata** may be often recognised by its naked-eye appearances, but a microscopic examination should be made in every case. Some scales from the edge of the ring should be removed with a blunt scraper, which has been previously moistened with a little liquor potassæ. These are put under a cover glass on a slide with a few drops of the potash solution, and examined with a quarter-inch objective. The mycelium threads are abundant, but the spores few. The former are long, delicate rods, branching here and there, and broken up into sections of different length.

The treatment of tinea circinata is simple as it is satisfactory. A single painting of the affected surface with tincture of iodine often suffices to kill the fungus, which, however, may require the stronger liniment of iodine, or even a blister. If an ointment be preferred, one composed of half a drachm each of pure carbolic acid and of salicylic acid to the ounce of vaseline may be worn continuously on lint beneath oil-silk. Should the patch be inflamed and pustular, the inflammation should be subdued with soothing lotions, as calamine or weak boracic acid. The writer has found a solution of salt and hyposulphite of soda āā. ʒ ij., with a drachm of glycerine to an ounce of water, a good application for these cases, especially when the disease has attacked the face.

2. **Tinea sycosis,** ringworm of the beard or eyebrows, is usually of the large-spored variety, and most common in young male adults. It is often contracted at the barber's, either from an infected razor, or more probably from an infected lathering brush. It begins as a small, red, more or less scaly spot, in the centre of which is a hair. This spot spreads, and sometimes forms a ring. At length many hairs are involved. Their follicles inflame and form numerous pustules. As a rule, beard ringworm is extremely chronic, disfiguring, and rebellious to treatment. It is often the seat of severe itching, and sometimes of a diffuse eczema, with brawny thickening of tissues. It may end in scarring and permanent baldness. Its recognition usually presents little difficulty, but an examination under the microscope should always be made. In that way it may be separated from ordinary non-parasitic folliculitis or eczema of hairy parts. Pathologically, tinea barbæ is an infective folliculitis and perifolliculitis, set up by the presence of the trichophyton. It is characterised by its attacking the hairy parts of the face, by its loosening of hairs, by its "lumpy" swellings, by its steady spread, and by the early and constant presence of pus. At the same time there is no serous discharge or "weeping."

Under the microscope the hair, treated with liquor potassæ, usually displays short-jointed threads in its substance. Sabouraud calls this endothrix, and says parasitic sycosis is always due to a large-spored variety derived from the horse. It seems likely, however, that the hairy parts of the face may be attacked by several varieties of ringworm. The diseased hairs become loose, and may be easily pulled out. For examination it is best to take a hair from the spreading edge. Prolonged soaking in the potash solution is sometimes needed to display the fungus.

The method of staining the fungus is given by Dr. Adamson[1] as follows: "(1) 5 per cent. to 10 per cent. solution of caustic potash on the slide, ten minutes to thirty minutes. (2) Wash in 15 per cent. alcohol in water. (3) Dry on the slide, and, in the case of scales, fix by passing through the flame. (4) Stain in gentian-anilin-violet (made in the usual way by the addition of a few drops of saturated alcoholic solution of gentian violet to anilin water), fifteen minutes to sixty minutes. (5) One to five minutes in Gram's iodine solution. (6) Decolorise in anilin oil, two or three hours, or longer. (7) Remove anilin oil by blotting-paper, and mount in Canada balsam." When the specimen has been soaked for some time (fifteen to thirty minutes) in the potash, the allowance of time for staining and for decolorising will be shortened and *vice versâ*.

If treatment be steady and persistent a cure may be predicted sooner or later, even in obstinate cases. The disease is often extremely chronic, and apt to recur after apparent recovery.

**Epilation** is useful, both as a means of getting rid of the fungus and of releasing pent-up pus. It should be carried out daily over a small area. The affected hairs are seized two or three at a time with a broad blunt forceps and extracted. The operation is almost painless, and may be repeated, say, over half a square inch every other day. The plucking of hair should extend to apparently sound parts around the patch.

**Shaving** the scalp affords valuable help to the surgeon in various ways. Thus, it disposes at once of a large quantity of the fungus. It enables remedies to reach the deeper seats of the mischief. It discloses the extent of the ringworm and the progress of the case. It should be carried out once a week, and washing the scalp had perhaps best be avoided altogether unless the head be previously shaved.

**Parasiticide.**—The main obstacle to cure is the difficulty of bringing the parasiticide into contact with the fungus. One of the best of ointments is composed of pure acid carbolic and salicylic acid, each half a drachm, to the ounce of vaseline. This may be rubbed into the patches with a stiff brush, which can be made by cutting short the bristles of an ordinary penny painter's brush. Chrysarobin half a drachm or a drachm to the ounce of vaseline, may be tried, but always with the greatest caution, for if the patient get a little of the drug in his eye it may cause severe suffering. Morris recommends a lotion of salicylic acid, five to twenty grains to the ounce of chloroform, applied every night. He says, however, that if the application is to succeed, no fatty substance must be used at the same time. A similar caution extends to iodine and blistering fluid. A good plan is to rub the shaved scalp with pure turpentine for three or four minutes, until the child cries out. The head is then washed with carbolic acid soap—dried, and painted over with tincture of iodine. This process may be repeated daily for a fortnight. Another plan has been found by the writer to answer well in chronic and intractable cases. After rubbing the head with turpentine, as above described, it is painted over with a solution of biniodide of mercury in spirit of the strength of 1 in 500. Each night the head is packed with lint soaked in the following lotion, and covered in with oil silk or a rubber bathing cap: ℞. Sod. chlorid.; sod. hyposulphit, āā. ℥j.; ol. sanitatis, ʒij.; aq. @ ℥x.; m. fat. lotio. Sign.: Apply on lint. When the head shows much improvement, an ointment of corrosive sublimate will often hasten matters in a remarkable way. It may be made thus: ℞. Hyd. perchlorid, grs. ij.; ac. salicylic, grs. xx.; adip. benzoat, ℥j.; m. ft. ung. Sign.: To be well rubbed into affected parts.

Many other methods have been advocated, but the foregoing should be enough to cure most cases in a few months. However, there will always be a certain margin that will resist treatment for years. When the disease has drifted into this chronic condition much good may often be done by stimulation with such applications as croton oil, coster's paste, liniment of iodine, strong acetic acid, or blistering fluid.

The application of iodine, it may be noted, has some practical value, because it stains the affected hairs a deep colour, and thus enables one to note the extent of the disease.

**Preventive Treatment.**—Reinfection of the patient from the cap may be prevented by wearing inside the head-covering a movable lining, which can be changed daily. If of paper, it should be burnt; if of linen, it can be sterilised by being placed in boiling water, or by being wrapped in paper and baked in an oven. Caps that have been worn without such a protective lining should be similarly disinfected in an oven, and, if covered with thick brown paper, will not be damaged. Brushes, no doubt, convey a great deal of infection to other persons, and reinfection to the original owner. They should not be used at all for ringworm patients; and, if infected, should be soaked for many hours in strong carbolic acid solution (1 in 20), and well washed with Hudson's or other strong scouring soap. All personal washing should be conducted with great care. No two children, for instance, should be allowed to wash in the same water. Neither should they use the same towels, sponges, or washing flannels. Then, again, one pillow should be kept strictly for the patient's use, and either be covered with a piece of linen that can be scalded, or have a fresh pillow slip every few days. All these points require special attention in schools. In such places, whether there is ringworm or not among the scholars, all towels should be carefully numbered and hung on separate pegs, so that every child may have his own towel. The same should be done with hats, combs, brushes, and washing flannels, and a separate basin be provided for each individual.

From what has been said it will be plain that the key to success in the future in dealing with this highly infectious disease lies in isolation. No child should be allowed to go to school who is suffering from the complaint. Moreover, Board and other schools require skilled inspection from time to time in order to weed out cases. That ringworm could eventually be banished from this country seems certain enough, if only the three common-sense rules of skilled inspection, rigid isolation, and special treatment were everywhere insisted upon.

[1] "The Permanent Staining of Ringworm Fungus." H. G. Adamson, M.D., "Brit. Journal of Dermatology," December, 1895, p. 377.

